# Acknowledgment to the Reviewers of *Veterinary Sciences* in 2022

**DOI:** 10.3390/vetsci10020068

**Published:** 2023-01-18

**Authors:** Veterinary Sciences Editorial Office

**Affiliations:** MDPI AG, St. Alban-Anlage 66, 4052 Basel, Switzerland

High-quality academic publishing is built on rigorous peer review. *Veterinary Sciences* was able to uphold its high standards for published papers due to the outstanding efforts of our reviewers. Thanks to the efforts of our reviewers in 2022, the median time to first decision was 18 days and the median time to publication was 42 days. Regardless of whether the articles they examined were ultimately published, the editors would like to express their appreciation and thank the following reviewers for the time and dedication that they have shown *Veterinary Sciences*:
Ad De GroofKatalin BuriánÁdám DeákKatarzyna Buńkowska-GawlikAdarsh K. GuptaKatarzyna Kliczkowska- KlarowiczAdel PezeshkiKatarzyna Ropka-MolikAdolfo Maria TambellaKatarzyna TajchmanAdriana AnitaKate TsaiAdriana CortezKaterina RoubalovaAdriana FerlazzoKatharina L. LohmannAdriana TrottaKatherine PflaumAdriana VinhasKátia Cristina PinelloAgnieszka Noszczyk-NowakKatia PinelloAgnieszka OrkuszKatja Mertens-ScholzAgnieszka WnukKatja SteigerAgustín Olmedo-JuárezKatsuhiro MatsuuraAhmad Jawad SabirKatsuki TsuchiyamaAhmed ElbestawyKatsunori OkazakiAhmed GalalKaushi S.T. KanankegeAhmed Neamat-AllahKazue OhishiAhmed NoreldinKazuhiko OchiaiAhmed S. MandourKazumi ShimadaAhmed SamyKazutoshi NishijimaAijun SunKegan Romelle JonesAitor Fernandez-NovoKeila Y. Acevedo-VillanuevaAjit SinghKelse Tibau De AlbuquerqueAkbar NawabKemal Tuna OlğaçAkira MatsudaKenji KutaraAkira YabukiKentaro YamadaAlan J. ConleyKevin KantonoAlan TilbrookKezong QiAlberto ElmiKhizar HayatAlberto EspíKhristine Joy C. AntiguaAlejandro A. TapiaKim BlasdellAlejandro J. Marquez-LaraKiwamu HanazonoAlena PechovaKiyonori KawasakiAlessandra Marnie Martins Gomes De CastroKlaudiusz SzczepaniakAlessandro FiorettiKlaus H. HoffmannAlessandro GuerriniKo-Hua TsoAlessandro PoliKonstantinos ArvanitisAlessandro SammarcoKonstantinos KoutoulisAlessandro SpadariKristen BradyAlessandro VastoloKristene R. GedyeAlessio BortolamiKristjan BregendahlAlessio PaladiniKrisztián BaloghAlessio PieriniKrystyna OlczykAlexander A. SermyaginKrzysztof GrzymajloAlexander KlothKrzysztof RypułaAlexander KrivoruchkoKrzysztof TomczukAlexander MottKurt KotrschalAlexander RuchinKwangwook KimAlexandra C. GreenKwonil JungAlexandra GambaryanKyle McLeanAlexandra I. GallerKylee Jo J. DubersteinAlexandra WealleansKyohei FurukawaAlexandre M. PinheiroKyung-Woo LeeAlexandre SilvaLabrini V. AthanasiouAlexey RuchayLakamy SyllaAlfredo Silveira De BorbaLars Erik RuudAli WahdanLászló FodorAlice BonomiLaura B. HeilenAlice MicheluttiLaura BenitezAlireza SeidaviLaura Giuseppina Di PasquaAlison SmallLaura HuberAlla SplichalovaLaura Montoro DasiAllison C. VilanderLaura Sujo-MontesAlri PretoriusLaure GatelAmanda K. Lindholm-PerryLaurent LocquetAmanda K. Warren-SmithLaurie ConnorAmanda WallerLavanya GoodlaAmandine DrutLavinia ChitiAmar ParvateLavinia CiucaAmelia Ramon-LopezLawrance ChandraAmer AlićLeena AwawdehAmin SayyariLeila AbdelhamidAmir-Babak Sioofy-KhojineLeonard LauriaultAmit VikramLeonidas LeontidesAmlan Kumar PatraLeonor DelgadoAmrita SarkarLeopold Slotta-BachmayrAmy JohnsonLeslie IrvineAmy MacNeillLetizia FiorucciAmy MillerLiang ChenAna AmaralLicia PantanoAna BotelhoLidia Lasecka-DykesAna BracarenseLihui ZhuAna C. A. Pereira De Mira GeraldoLilia GutierrezAna CanadasLilian SosaAna Catarina TorresLin Lee-ShuanAna Cláudia Correia CoelhoLin WalkerAna Claudia FrancoLina María Martínez-LópezAna Cristina FerreiraLinde GilleAna Del CerroLinus OlsonAna Isabel Fraguas-SánchezLiqian ZhuAna Isabel Rocha FaustinoLíriahiromi Hiromi OkudaAna Martí-De OlivesLi-Ting ChengAna Roca-FernándezLivio GalosiAna RostaherLluis FerrerAna Shek-VugrovečkiLongchao ZhangAnastasia DiakouLorenzo DomenisAnastasia KirillovaLorenzo TiduAndjelija Ivkov DzigurskiLori KoganAndra-Elena EnacheLou KimAndré Felipe StreckLouise Van Der WeydenAndré L.V. ZoppaLuca SardiAndrea CordaLuca StucchiAndrea Dorothea EllisLuca VillaAndrea GarmynLucia CarlucciAndrea MarchegianiLucia LazarowskiAndrea Micke MorenoLuciana MandrioliAndrea PalombieriLucilla IacuminAndrea ZatelliLudovic MartinelleAndreas VernunftLudovic PelligandAndreia GarcêsLudwig E. HoelzleAndrew ByrnesLuigi NavasAndrew CookeLuigi RosatiAndrew KnightLuís CardosoAndrew NiehausLuis J. Ezquerra CalvoAndrew PetersLuis Miguel De PablosAndrew WallerLuisa RambozziAndrew WoodwardLuisa Vera MuscatelloAndrzej JarynowskiLukas SchwarzAndy GreerLuke Mangaliso DuncanAneliya MilanovaLuo-Yang DingAneta AndronowskaLuzia Helena QueirozAneta NowakiewiczLuzia RastAngel CataldiLynn M. PezzaniteAngela M. Bosco-LauthLysimachos G PapazoglouAngela M. Garcia SanchezM. Katie SheatsAngela MartinsM. PolinasAngélica Villarruel-LópezMaarten S. HollemansAngeliki KatsafadouMaciej GajęckiAngelo QuarantaMaciej JaneczekAnil VermaMadhavi L. KakumanuAnja JoachimMagdalena Agata GarncarzAnja KiparMagdalena Chottova DvorakovaAnn HemingwayMagdalena MaselloAnna AspánMagdalena ŻmigrodzkaAnna BeniniMakoto HasegawaAnna Duda-MadejMakoto OzawaAnna GajdaMalgorzata DominoAnna HrabiaMalgorzata GrzesiakAnna KasprzykMałgorzata Kotula-BalakAnna Lenart-BorońMalgorzata OlejnikAnna Luiza Facchetti Vinhaes AssumpçãoMandy PatersonAnna OrtuñoManiscalco LorellaAnna PapachManjusha ChintalapatiAnna StępniowskaManuel BelliAnna Szczerba-TurekManuel ChamorroAnna WójcikManuel García-HerrerosAnnamaria PassantinoManuela CrastaAnne ConanManuela GizzarelliAnne FünfhausMara BagardiAnne LichtenwalnerMarc GirondotAnne RousselMarc HeyndrickxAnneke MorescoMarcelo Cavenaghi Pereira Da SilvaAnnette McCoyMarcelo De Las HerasAnthony M. CarterMarcin ArciszewskiAnthony SchryversMarcin Bartłomiej ArciszewskiAnthony William ColemanMarco PorporatoAntoinette Simpah Anim-JnrMarco TecillaAnton GarciaMarcos Veiga Dos SantosAntonella GallucciMarcos ZeddaAntonella PuggioniMarcus FelicianoAntonella VoltaMardik LeopoldAntonín PřidalMarek GaworskiAntonio BuendíaMargret L. CasalAntonio CarminatoMaria Adela Valero AleixandreAntonio FernándezMaria AlbrizioAntonio GiulianoMaria Angels CalvoAntonio Gonzalez-BulnesMaría Buendía-AbadAntonio MolloMaria C. KantereAntonio SantanielloMaria C. VeronesiAnuj AhujaMaria Carmela PisuAphrodite I. KalogianniMaria D. FernandezApostolos D. GalatosMaria DaskalakiArif SiddiquiMaria De OliveraAris PourlisMaría Dolores Llobat BordesAristotelis LymberopoulosMaria Dos Anjos PiresArkadiusz MiazekMaria Elena MartinoArlene McDowellMaria Filomena CaeiroArnaud Van WettereMaría Florencia ViannaArul ChandranMaria FranciosiniArun RajamohanMaria Giovanna CilibertiArvind SharmaMaria Grazia CappaiAsha M. MilesMaría Jesús Serrano AndrésAshenafi F. BeyiMaria João FradinhoAshley FowlerMaria João SoaresAspinas ChapwanyaMaría José Cubero PabloAtsuki NaraMaría José García IglesiasAtsushi KatoMaria José Ranzani-PaivaAttila ZsolnaiMaria KantereAttilio CorradiMaria Luisa DettoriAugusto CarluccioMaria Luisa Pinna ParpagliaAulo ManinoMaría Martín-CuervoAura GabrielMaria MenshakovaAyyanar SivananthamMaria Montserrat Rivera Del ÁlamoBajram BerishaMaria RizzoBao YuanMaria SkrzyszowskaBarbara BrunettiMaria Teresa Armúa-FernándezBarbara CanonicoMaria Zelinda RomanoBarbara De AngelisMaría-Luz GarcíaBarbara De MoriMarian FlisBarbara KluppMariano CarossinoBarbara LamagnaMariano Francesco CaratozzoloBárbara Martín-MaldonadoMarie Christine CadierguesBarbara MoroniMariko OkamotoBarbara SchöningMarília TakadaBarbara SzczepankiewiczMarilisa NovaccoBartosz PawlińskiMarin KovačićBas B. Oude MunninkMarin TortiBeata WodeckaMarina DiioiaBeatrix AlegreMarina NeofytouBecky HessMario CinoneBee Kang TanMario D'IncauBenedict KjærgaardMario RothbauerBenito Soto-BlancoMariola BochniarzBenjamin CullMarion MuchaBernard RoelenMarios SpanakisBernd HoffmannMarius MasiulisBertrand L. DeputteMarius PenteaBettina WollankeMarius ZahanBiljana BufanMark A. BrownBishoy Y.A. El-AaragMark A. SuckowBo WangMark CraigBoyko B. GeorgievMark F. WiserBożena KiczorowskaMark FarnworthBrandy BurgessMark HansBrett BlighMark MoseleyBrett DeGregorioMark RishniwBrian EllisMark StewartBrian FlesnerMarkku SaastamoinenBrian MurphyMarko HaloBrigid Victoria TroanMarko R. CincovićBrigitta WichertMarko SamardžijaBritta VidoniMarlies BallegeerBruna VisniauskasMarta CanutiBrunella RestucciMarta DecBryony S. TuckerMarta KankoferCaio Seiti TakiyaMarta NardiniCalin Mircea GhermanMarta Waliszewska-ProsółCallum George DonnellyMartha LarsonCamilo Hernández-AvilésMartin BachmannCao ShinuoMartin CeballosCarina De Fátima RodriguesMartin O. FurrCarina ReuscherMartina ColomboCarlo V. CitterioMartyn JeggoCarlos Alfredo Sandoval CastroMarwan OsmanCarlos Daniel Gornatti-ChurriaMaryAnn G. RadlinskyCarlos Eduardo Fonseca-AlvesMary-Louise PenrithCarlos GrauMarzena Rola-ŁuszczakCarlos H RomeroMasaaki TaniguchiCarlos Iglesias PastranaMasahiro YasudaCarlos MarcuelloMasami MorimatsuCarlos MelianMasaomi KurokawaCarlos Sandoval-CastroMasashi KawamiCarlos ViegasMasashi MaitaCarlotta GirominiMashkoor MohsinCarmela ValastroMasmudur M. RahmanCarmen IscaroMassimiliano De PaolisCarmen JerryMassimiliano TursiCarmen Ledesma-FelicianoMatej MurinCarmen SolcanMateusz MikiewiczCarmine MerolaMatilde LombarderoCarolina Balao Da SilvaMatthew ScottCarolina Hernández-LaraMatthew StrubCarolina TamargoMatthias ContzenCaroline GriffittsMaureen MacNamaraCaroline HahnMauricio Javier GiuliodoriCaroline RitterMauricio L. CanalsCaterina SquillaciotiMazeyar Parvinzadeh GashtiCatherine A. GordonMd. Bashir UddinCathryn SparksMeerambika MishraCathy McGowanMeghan T. RamosCátia MarquesMelanie DobromylskyjCecile Bienboire FrosiniMelvin E. KlegermanCécile ClercxMenelaos A. LefkaditisCecilia Dall'AglioMenno HolzhauerCecília R.C. CaladoMercedes Fernández-EscobarCeferino LópezMichael BetteCengiz GökbulutMichael DaviesChadi EidMichael E. BarnesChaiyapas ThamrongyoswittayakulMichael E. HumeChalong WachirapakornMichael FlytheChanghyun RohMichael HässigChang-Jun ZengMichael HerrtageCharles BrownMichael J. GladeCharles Odilichukwu R. OkpalaMichael L. HessCheng TangMichaela A. BotiCheng-Yih HongMichał BulcChen-Si LinMichał DzięciołChensiang NgMichał JankChiara Del PreteMichal JurasekChiara GarbarinoMichał MajewskiChiara GiudiceMichela PuglieseChiara Grazia De CiucisMichelle GarciaChiara SeminatiMifumi KawabeChristian HanzenMiguel A. AparicioChristian HidalgoMiguel MatosChristian OrtaldaMiguel MelladoChristina MontalbanoMiguel QuaresmaChristina R. Wilson-FrankMihaela NiculaeChristof Albert BertramMihai MusteataChristophe R. CasteleynMike McGrewChristopher RiggsMikio HayashiChrysanthi VoyiatzakiMiles TheurerChun-Ren WangMing YangCinzia BenazziMingmin LuCiprian OberMinhua SunCiro InvernizziMinhui GuanClaire Beaudu-LangeMircea Ioan PopaClaire SimeoneMirela ImreClara MarínMirela J. Wolf-BacaClaudia EleniMirja K. HuhtinenClaudia FernandesMiroslav RadenkovicClaudia GarridoMirosław MichalskiClaudia Maria TucciaroneMisako KonishiClaudia Pérez-MartínezMitsuhiko AsakawaClaudia RificiMo SalmanClaudia SpadavecchiaMohamed ZeineldinClaudio De LiberatoMohammad DelghandiClaudio GenchiMohammed GagaouaCorinne Marois-CrehanMohit Kumar JollyCraig Robert ReinemeyerMohsen M. RamadanCristian Manuel Suárez-SantanaMolly NicodemusCristian TorresMonika Hułas-StasiakCristina López CadenasMonika OlechCristina Maria PonepalMonika Proszkowiec-WeglarzCristina MesquitaMonika ReissmannCristina P. VicenteMonika RinderCristina QueirogaMonika Szymańska-CzerwińskaCristina SobacchiMonika Wensch-DorendorfCristina VercelliMonique MancusoCyril HrnčarMorgan BrisseCyril RoyMorten Dam RasmussenDan HellerMorten RasmussenDanica PollardMotoko MorimotoDaniel BogemaMuhammad FarooqDaniel BruggerMuhammad IkramDaniel EllederMuhammad Sohail ZafarDaniel MongioviMuhammed Shafeekh MuyyarikkandyDaniel Moura De AguiarMuhammet KarakavukDaniel Sanchez-MasianMujahid Ali ShahDaniel W. NixonMyron ChristodoulidesDaniela DenkNadine BaumgartDanielle B. GrahamNadja SigristDanijela ČerneNaga Venkata Sabarinath NeerukondaDanijela Horvatek TomicNagendra Kumar KaushikDaphne T. LianouNagendra Nath BarmanDarci Moraes Barros-BattestiNagendrakumar Balasubramanian SinganalurDaria MurawskaNagy IstvánDaria V. EvsyutinaNaoko YanagisawaDariusz M. StasiakNatalia FantonDariusz MikulskiNatalia KasicaDariusz PiwczyńskiNatalia SiwińskaDavid A. ComerfordNatalie LenardDavid A. MacLeodNathalie PryimenkoDavid FerreiraNathalie Wissink-ArgilagaDavid KilroyNausikaa DevriendtDavid N. McMurrayNektarios D. GiadinisDavid RotsteinNektarios KoufopoulosDavid SteffenNenavath Gopal NaikDavide BorroniNeoklis ApostolopoulosDavide CossuNeringa AlisauskaiteDavide De LorenziNéstor SepulvedaDean HendricksonNicholas DodmanDebra HickmanNicola DecaroDeepani FernandoNicola ZizzoDennis E. JewellNicolai HildebrandtDennis L. SchmittNicole Burdick SanchezDenny BöttcherNidia Arechiga-CeballosDhananjay YadavNikki KellsDhruv DesaiNikola HodkovicováDiana FanelliNikola KezicDianjun CaoNikola M. PuvaĉaDiego MartinezNikola MetodievDiego PiantedosiNikolaos DiakakisDiego Rodrigo MocholiNil PandeyDiky RamdaniNils Petter KjosDilip Kumar SwainNina WolfrumDimitris A. GougoulisNisar AhmadDimitris DimzasNishida TakehiroDirceu RamosNobumichi KobayashiDmitry VlasovNobuo SasakiDomenico BrittiNolberto ArismendiDominik ŁagowskiNorihiko ItoDonald J. MeutenNuno Henrique FrancoDongdong ZhangNuno V. BritoDongmin ZhaoNussieba A. OsmanDorota WojniczOana-Maria BolduraDorota ZielińskaOksana ZininaDragan MilicevicOlga MitjanaDuccio PanzanniOlga PawełczykDuyang GaoOlga Szaluś-JordanowDylan M. JohnsonOlga Witkowska-PilaszewiczEduardo CostaOlga Zorman RojsEduardo De MercadoOlimpia LaiEduardo Gutierrez-BlancoOlivera DjuragicEdyta PasickaOlivia Rodriguez-MoralesEgil Andreas Joor FischerOluwatobi KolawoleEirini FragkiadakiOmar Antonio Gonzales VieraEkaterini MoschopoulouOndrej HanušovskýEkramy SayedahmedOri PomerantzElaine NortonOrman L. SnyderEleanor WatsonOwais A. KhanElena BiasibettiPajor FerencElena De FelicePalak PatelElena GonellaPaloma Jimena De AndrésElena NikitkinaPam Dachung LukaElena TchetinaPamela MurisonElena TrombettaPanagiotis KarakaidosEleni Georgios KatsogiannouPanagiotis SimitzisEleonora NicolaiPanagiotis TassisEliana Ruth SteinbergPanakova LuciaElias P. PapapanagiotouPanchan SitthicharoenchaiEline WydooghePankaj DhakaElisa ManzocchiPaola Dall’AraElisa MazzottaPaola GalluzzoElisabeth Lieber-TenorioPaola ModestoElisabeth MathijsPaola StraticòElisabetta GiudicePaolo CapozzaElisabetta ManaresiPaolo Dalla VillaElisabetta ManualiPaolo De GirolamoEliza CurnowPaolo PastorinoElizabeth A. ClemmonsPaschalitsa TryfinopoulouElizabeth DavisPatricia A. BoleyElizabeth SpargerPatricia Mora-MedinaEllen RoelfsemaPatrick ButayeEloísa SevillaPatrizia LicataEl-Sayed Abdel-WhabPaul BatesElżbieta JancewiczPaul BloomEmad Al-DujailiPaul HonessEmerson José VenancioPaul RossiterEmilie RecoulesPaul W. C. WongEmir HodzicPaula Rozo-LopezEmma JohnsonPaulo CarneiroEmmanouil I. PapadogiannakisPaulo Fantinato NetoEmmanuel SerranoPawel BieganowskiEmmanuelle BourneufPaweł SolarczykEmoke PallPawel ZmoraEnrico AllevaPedro GonçalvesEnrico BrugneraPengbiao XuEnrico DesideriPetar KostešićEnrico GugliandoloPeter CakebreadEnrico PavoniPeter GrovesEraldo Sanna PassinoPeter Hendrik KookErasmia SidiropoulouPeter HillEric CoïcPeter KnightEric Free EgelundPeter MansellEric MillerPeter PascoeEric R. BurroughPeter TozerErin L. LegackiPetra BandeljÉriton Egídio Lisboa ValentePetronella KettunenErkan CurePhilip J. SeddonEsmeralda DelgadoPhillip Anthony MooreEsterina FazioPierangelo MorettiEsther MorenoPierre PicavetEugênio Gonçalves De AraújoPieter CornillieEva CunhaPietro BadagliaccaEva Maria Perez MerinoPinna StefaniaEva SpadaPiotr CzyzowskiEva StrakováPiotr JurkaEva VoslářováPiotr KuropkaEvelien DierickPoonam SharmaEvelyn GalbanPremranjan KumarEvie MelanitouPriscilla F. GerberEwa Joanna GodzińskaPrzemysław KołodziejEwa LaskowskaQi YanEwa TomaszewskaQianqian ZhaiF.G. (Frank) Van SteenbeekQing YangFabio CastagnaQinghua QiuFabio De RensisQingmei XieFabio MarinoQuynh Anh LeFabio OstanelloRachel GilroyFadis F. MurzakhanovRachel PalinskiFanfan ZhangRadojica DjokovicFang TangRadu Ionel NeamtFatih HatipogluRadu-Andrei BaisanFatima El KhalloufiRafael Julio Macedo BarragánFatma EldemeryRafael R. GopeguiFederica BrunoRafał CiaputaFederica Del SignoreRafal KretowskiFederica Di CesareRafał PingwaraFederica LoiRafał SapierzyńskiFederico ArmandoRaffaella CoccoFederico CorlettoRakesh BhaskarFederico InfascelliRalf BlankFelice AdinolfiRalph E. HamorFelice CrocettoRalph Eric Thijl VanstreelsFelix ChanRamón ArmengolFelix SchmittRamunas AntanaitisFernanda Da Cruz LandimRansom BaldwinFernanda SeixasRasa BernotienėFernanda Viana CabralRasa NainieneFernando Capela E SilvaRashid ManzoorFernando FerreiraRaul Alegria-MoranFernando Gonzalez-UarquinRavindra DeshpandeFernando José Figueiredo Agostinho D’Abreu MendesRebecca WindsorFernando López-GatiusRebeka ZsoldosFidel San RománRebekah R. HeltonFilippo FratiniRegiane Maria Tironi De MenezesFirdoos Ahmad GogryRegula Bettschart-WolfensbergerFirdous A. KhanReiichiro SatoFloryne O. BuishandReinaldo Dos SantosFoteini SamartziReinhard ErtlFranca AnglaniRemo LobettiFrancesca AbramoRenata FreitasFrancesca De FalcoRenata PiccininiFrancesca De SantisRenato CeccherelliFrancesca Del ChiccaRene KadenFrancesca GrippiRenée Laufer-AmorimFrancesca Paola NoceraRicardo Andrade ReisFrancesca ParisiRicardo F. StrefezziFrancesca Romana MassacciRicardo LucenaFrancesco BertiRicardo Nalda-MolinaFrancesco CollivignarelliRiccardo RinnovatiFrancesco DefilippoRiccardo TelliniFrancesco MacrìRiccardo ZelliFrancesco PriscoRichard BowenFrancisco A. ArrebolaRichard G. HarveyFrancisco Gerardo Véliz DerasRichard JonesFrancisco Javier Alvarez-MartinezRichard MalikFranjo MartinkovićRichard UwieraFrank Francis A. DrummondRifat Ullah KhanFrans N J KooymanRiley ThompsonFranziska KühneRina VerdiglioneFraser HillRita Payan-CarreiraFrederic BeugnetRobert Andrew CushmanFrederico VieiraRobert BrosnanFrederik PauwelsRobert Cristian PurdoiuFulvia BoveraRobert DaileyFun-In WangRobert E. MeyerFusheng SiRobert GłogowskiGábor MátisRobert J. Van SaunGabriel Cuevas RamosRobert KammererGabriela Chavez-CalvilloRobert L. LarsonGabriela HirsbrunnerRobert PasławskiGabriele C. GhisleniRobert RekawieckiGabriele GerardiRobert Yung Liang WangGabriele RossiRoberta Ferro De GodoyGaelle GonzalezRoberta PeregoGail Miriam MoraruRobertas JuodkaGalli Gabriela MiottoRoberto Sánchez SánchezGarikoitz AzkonaRoberto TamburroGarrett MullenixRobin NicholasGáspárdy AndrásRobyn HallGayeon WonRobyn HudsonGema Martínez-NavarreteRodrigo Muíño OteroGene M. PestiRodrigo NovaGenxi ZhangRodrigo Rosario-CruzGeorge AnifandisRomeo Theodor CristinaGeorge FthenakisRonald S. JonesGeorge KazakosRong WangGeorge MichailRosalia CrupiGeorge ValiakosRosanna MarinoGeorgios BatziasRuangyote PilajunGeorgios MantziarasRuifang WeiGeorgios PapadopoulosRuizhi HuGeorgios TsousisRussell S. FraserGerald Fritz SchusserRuth Lindizyani MfuneGerardo FatoneRyan TroyerGert NiebauerSabrina WangGert VenterSachiko Miyagawa-TomitaGheorghe DărăbuşSaeid HosseinzadehGiampaolo ColavitaSai-fu FungGianluca FichiSalvatore DesantisGianvito LanaveSam RidgwayGiliola SpattiniSamuel HockerGillian EastwoodSandra AungierGina-Cecilia PistolSandra BrancoGioele CapilloSandra FoltinGiorgio FedrizziSandra HöglerGiorgio SmaldoneSandra SoutoGiovanna Lucrezia CostaSantiago PeraltaGiovanni AsteSantina Di BellaGiovanni CiliaSanto CristarellaGiovanni Della ValleSantosh DhakalGiovanni FormatoSara Andrés-LasherasGiovanni GhibaudoSara BellucoGiovanni NieroSara BusechianGiovanni PallioSara DamianoGiovanni Pietro BurraiSara FuochiGiovanni PoglayenSara MangiaterraGiovanni RomitoSara MarquesGiulia BallottaSara SechiGiulia GuerriSarah C. WoodGiulia MorgantiSarah L. MasonGiulia PignataroSarah MorrisonGiulia SienaSarit PalGiuliano BorrielloSatoshi KawakamiGiulio SeveriSauve FredericGiuseppe MarruchellaSaw BawmGiuseppe PassantinoSayed Haidar Abbas RazaGiuseppe PiccioneScott ReidGiuseppe SpinellaSebastián Demyda-PeyrásGlaecir Roseni Mundstock DiasSejian VeerasamyGlenys NobleSelvaraj PavulrajGloria ArriagadaSelwyn HeadleyGopinath KrishnasamySepideh HosseiniporghamGotthold GäbelSerena CavalleroGrace CarrollSerena ReggiGrace G ZhouSerenella SilvestriGrant DewellSergio Ángeles CamposGreg A. VicinoSergio Gastón CaspeGreg S. BaxterSergio MiglioreGregory JohnsonSergiu FendrihanGregory TawaSeunghyung LeeGreta FoianiShahin TajeryGreta Veronica BerteselliShahista NisaGrulke SigridShaimaa SelimGrzegorz WoźniakowskiShangzhe XieGuangbo GeSharma SachinGuangyu LiSharmila GhoshGuangzhao LiShashank PandeyGudrun StefánsdóttirShashwati MathurkarGuido GrilliShaying ZhaoGuido Ruggero LoriaShengguo ZhaoGuillaume FichesSherry ShuGuillermo Moreno-SanzShihlung ChenGuixue HuShilpi JainGuobo QuanShimi MeleppattuGuoliang LiShinobu MatsuuraGustavo DelhonShubhagata DasGwendolen LorchShuen-Ei ChenGyanendra GongalShulin FuHachung YoonShuo YanHai LinShu-Yin WangHaitao ShiShyi-Kuen YangHaiyan SunShyunji HayashiHanchuan DaiSiavash Salek ArdestaniHani Ba-AwadhSibylle KneisslHan-Jun KimSilvia BelliniHanna KuligSilvia CeroliniHanna LindqvistSilvia DottiHannah ShawSilvia FacciniHans G. DrexlerSilvia FerroHan-Tsung WangSilvia Michela MazzolaHao ChenSilvia PreziusoHao LiSimon BertramHaoping LiuSimon CleggHarini SooryanarainSimon Peter MusinguziHarold SalantSimona CannasHatim I.K. AlibhaiSimona Di PietroHe ZhangSimona Kralova-KovarikovaHeather K. MoberlySimona SacchiniHéctor Raymundo Vera ÁvilaSimona SagonaHee Bok ParkSimona TafuriHeike Aupperle-LellbachSimona VincentiHeike U KöhlerSimone BelliHeinz BernhardtSimone De BrotHelder Patrício NunesSimonetta CitiHelder QuintasSladjana RakitaHelena RylanderSławomir ZduńczykHenri Georges Michel Justin BertrandSofia Alves-PimentaHenrik RönnbergSofia Costa-de-OliveiraHenry MunyandukiSofia LamasHerris MaxwellSohail QaziHikichi YutaSoma DashHilla ChenSong Toan TranHiroshi AsakuraSoo-Hyun SungHiroyuki AonoSouquan ZhangHitoshi MurakamiSowmya PoosapatiHong-Jiang WeiSpiri Andrea MonikaHoon JangStanisław DzimiraHossam El-Sheikh AliŞtefan Gregore CiorneiHsinbai YinStefan HobiHuafeng WangStefania PerrucciHuang YongStefanie VeraaHugo Arias-PulidoStefano LafranceschinaHui-Hua HsiaoStefano RomussiHussein Mohamed Elhusseiny Ali ElbayoumiStephane RetyHye Kwon KimStephanie A. ThomovskyHyeck-Soo SonStéphanie NoëlHyo-Sun KwakStephanie Stanelle-BertramHyun-Jin ShinStephen A. SmithIan SutherlandStephen ColeIbrahim BelalStephen FairweatherIbrahim ElsohabySteve HartIgor Y. IskusnykhSubarna BaruaI-Hsin SungSubramaniam SaravananIlaria PorcellatoSue DysonIlias GiannenasSue MurphyIlya M. TereninSukhvinder SinghIman Mehdizadeh GohariSunghyun YoonImtiaz RandhawaSunny S.K. ChanInês L. S DelgadoSupriyo RayInge LarsenSurekha ShridharInger AnnebergSusan DuckettIngrid LorenzSusan PatersonIngrid Reiter-OwonaSusana RemesarIngrid TonniSusanne BoroffkaIoan HutuSusumu MuroyaIoannis A. TsakmakidisSuzanne RogersIoannis S. PappasSvenja SpringerIoannis SavvasSybille D. ReichardtIonela HoteaSyed Azmal AliIrene NoceraSylvia García-BelenguerIrene SartiniSylwia ProchowskaIrene ValasiTadashi SanoIrenilza De Alencar NääsTadeusz StefaniakIrina Garcia IspiertoTaís Fukuta CruzIrina HeidTajana Trbojevic VukicevicIsaac Hyeladi MalgwiTakanori KooriyamaIsabel CasasTakashi HashimotoIsabel Loreto Barroso De CarvalhoTakehiro NishidaIsaia SymeonidouTakele ArgawIshmael Festus JajaTakeshi OhkuboIstván KissTakeshi OsawaItziar Chapartegui-GonzálezTakuichi SatoIulia-Cristina BagiuTakuya ItouIvan DimovTa‐Li LuIván Pastor FernándezTalita ResendeIvan PavlovićTamara BeckerIvana CabarkapaTânia MartinsIvo SirakovTanja E WolfIzabela JanusTansol ParkIzabela RaganTanushree DangiIzabella DolkaTapas MandalIzabella RządTara G. McDaneldIzhar Hyder QaziTarek MorsyJ Eduardo RicoTarique HussainJ. Alberto Montoya-AlonsoTaru S DuttJack H.Z. WuTasha Marie Santiago-RodriguezJackson Victor De AraujoTaya FordeJacqueline BoydTemple GrandinJacqueline KingTeresa Letra MateusJacques FontaineTeresa RochaJacques TheronTeresa SouthardJae‑Ik HanTereza Cristina CardosoJae-Ku OemTerrie M. WilliamsJaeson CallaTerry Ann ElseJaime SanchisTerry WhitingJake ShivleyThangavel RajagopalJalil Ghassemi NejadTheodoros NtallarisJames A. MarrsTheophanes LiatisJames BreretonThomas BlahaJames R. CrabtreeThomas C. WebsterJames T. KellyThomas GrönthalJan LadewigThomas HartingerJan S. SuchodolskiThomas SchermerhornJan StępniakTim SecottJan UdalaTimothy John MahonyJana SchäferTina KotnikJane M. MorrellTiong Kai TanJane ManfrediTomasz FlorowskiJane QuandtTomasz HikawczukJanez SalobirTomasz SadkowskiJanine A. DanksTomasz SzaraJanko SkokTomislav MikušJan-Peter BachTommaso VezzosiJarl BøgwaldTomohiko YoshidaJason B. PieperTomohiro YonezawaJason D. KeeganTomomi TakanoJason Devoy-KeeganTony GlausJason FarlowToshiaki AraJavier Blanco-MurciaToshiharu YamamotoJavier Gómez-ArrueTosso LeebJavier Ventura-CorderoTristan DarlandJean GuardTugay AyasanJean MukherjeeTurner H. SwartzJean-Marie GraïcUpendar GollaJeanne Brugère-PicouxUrs GigerJean-Pierre FrossardUrszula LisieckaJeffrey GilbertUte MackenstedtJegan IyyathuraiV M Krushnarao KottedaJennifer B. NagashimaValentina Virginia EbaniJennifer KetzisValeria AlbaneseJennifer RepacValeria GriecoJenny WilkinsonValeria TaurisanoJens EhmckeValerie E. RymanJens KrollValeska M. StephanJessica A. CanterVanner BoereJessica E. FellmethVasia S. MavrogianniJessica Maria AbbateVasileios PapatsirosJessica Suagee-BedoreVassilios DotasJesus A. BaroVassilis SkampardonisJèsus Maria Perez JimenezVassiliy TsytsarevJevrosima StevanovicVeerupaxagouda PatilJiagang TuVelmurugu RavindranJianbo ChengVicente EsteveJianing FuVictor LucioJianjun ZhaoVictor RiithoJiann-Horng LeuVictoria WatsonJianwei ZhouVijay IndukuriJianying GuoVijay K. SharmaJianzhu LiuViju Vijayan PillaiJiayue YanVinay ShivannaJieshi YuVincenzo CarcangiuJih Jong LeeVincenzo CicirelliJimenez Carolina PalaciosVincenzo TufarelliJingchun LiVioleta G. TruscaJinyuan ZhouVirginia Gamino RodríguezJiří HejnarVita RiškevičienėJiufeng WangVitalii TimofeevJoanna DąbrowskaVito BiondiJoanna GŁodekVitomir DjokicJoanna Klećkowska-NawrotVittorio SaettoneJoanna WojtackaViviana GrecoJoão Augusto FerreiraVladimir RajovićJoao Carlos Gomes NetoVolker SchmidtJoão CotaW. Jean DoddsJoao Henrique Moreira VianaWaldemar GrzegorzewskiJoão MesquitaWaldemar KazimierczakJoão Pedro BarbasWanda Olech-PiaseckaJoão QueirosWei-Dong LiJoaquim CarrerasWeisheng CaoJoaquim HenriquesWeiwei LiuJoaquín SopenaWen Tsan ChangJohan CaratyWenfeng LiJohann KoflerWenjun ZhuJohannes ReichWenlong ZhangJohannes SchenkelWesley Lyeverton Correia RibeiroJohn CarrWilliam Burton StaniarJohn McPhersonWilliam G. RyanJohn MundayWilliam PérezJohn O. OnukwuforWioletta BielJohn VoyerWojciech ŁopuszyńskiJohn WebsterWolfgang BäumerJonas HabelWolfgang WetscherekJonathan ElliottWon-je JangJongki ChoWoo-Jin SongJongseo MoXesús FeásJorge López-OlveraXianghong JuJorge R. KawasXiangpeng DaiJorgelina Di PasqualeXiao ChenJosé Alfonso AbeciaXiaolong KangJose C. SouzaXibi FangJosé Guadalupe Herrera HaroXin YangJosé Guilherme XavierXinheng ZhangJosé Luis Pérez-CastrillónXinran LiuJosé Manuel Jaramillo OrtizXu WangJosé María BlascoXuan LiJosé R. PinedaXudong SunJosé Raduan JaberYatta BoakariJose Sanchez-BetancourtYazavinder SinghJoseph BurrascanoYe JinJoseph ByaruhangaYexiang TongJoseph Cyrus ParambethYi YangJoseph LozierYifei LiaoJosilene Nascimento SeixasYihan XiaJowita Samanta NiczyporykYlva Cecilia Björnsdotter SjunnessonJoy ArcherYong GuoJuan Alberto CorberaYoshihisa HashiguchiJuan Carlos GardónYoshinao KuboJuan HernandezYosra A. HelmyJuan Hernandez GarciaYosra SoltanJuan L. HernandezYoussef AttiaJuan LiYouxiang DiaoJuan M. Guillen-MuñozYu-Chih WangJuan Manuel PerezYudai InabuJuan Manuel TeijeiroYuichiro HatanoJuan Miguel Soria GarciaYujie SuJuán MorgazYun MaJuan Pablo Perea-RodriguezYun ZhuJuan V. González-MartínYusheng LiangJudith C. KreutzmannYuting WanJuei-Tang ChengYuzuru IkedaJulia Steinhoff-WagnerYvette Nout-LomasJuliana CargneluttiZaisen WangJulie OldZalán HomonnayJulie ThomasZbigniew AdamiakJulio Alonso-PadillaZhengrong GuanJulio Vicente Figueroa-MillánZhenwei LiJun WuZhigang ZhuJuneo Freitas SilvaZhonghou WangJunqing GuoZiqiang ChengJuraj MajtanZoltán DeimJustina PradaZoltan M. VargaJyunsuke ShiraiZurisaday Santos-Jimenez


